# A mixed-methods exploration of attitudes towards pregnant Facebook fitness influencers

**DOI:** 10.1186/s12889-023-15457-6

**Published:** 2023-03-27

**Authors:** Melanie Hayman, Marian Keppel, Robert Stanton, Tanya L. Thwaite, Kristie-Lee Alfrey, Stephanie Alley, Cheryce Harrison, Shelley E. Keating, Stephanie Schoeppe, Summer S. Cannon, Lene A. H. Haakstad, Christina Gjestvang, Susan L. Williams

**Affiliations:** 1 Appleton Institute, School of Health Medical and Applied Sciences, Central Queensland University, Bruce Highway, North Rockhampton, Brisbane, QLD 4702 Australia; 2 Cluster for Resilience and Wellbeing, Appleton Institute, 44 Greenhill Road, Wayville, SA 5034 Australia; 3 School of Human Movement and Nutrition Sciences, The University of Queensland, Brisbane, QLD 4072 Australia; 4 School of Public Health and Preventive Medicine, Monash University, Wellington Road, Melbourne, Australia; 5 Department of Sports Medicine, Norwegian School of Sports Sciences, Ullevål Stadion Melbourne, P.O Box 4014, Oslo, VIC 3800 0806 Norway

**Keywords:** Social media influencer, Pregnancy, Exercise, Attitudes, Facebook

## Abstract

**Background:**

Exercise during pregnancy is associated with various health benefits for both mother and child. Despite these benefits, most pregnant women do not meet physical activity recommendations. A known barrier to engaging in exercise during pregnancy is a lack of knowledge about appropriate and safe exercise. In our current era of social media, many pregnant women are turning to online information sources for guidance, including social media influencers. Little is known about attitudes towards pregnancy exercise information provided by influencers on social media platforms. This study aimed to explore attitudes towards exercise during pregnancy depicted by social media influencers on Facebook, and user engagement with posted content.

**Methods:**

A mixed-methods approach was used to analyse data from 10 Facebook video posts of social media influencers exercising during pregnancy. Quantitative descriptive analyses were used to report the number of views, shares, comments and emotive reactions. Qualitative analysis of user comments was achieved using an inductive thematic approach.

**Results:**

The 10 video posts analysed were viewed a total of 12,117,200 times, shared on 11,181 occasions, included 13,455 user comments and 128,804 emotive icon reactions, with the most frequently used icon being ‘like’ (81.48%). The thematic analysis identified three themes associated with attitudes including [1] exercise during pregnancy [2] influencers and [3] type of exercise. A fourth theme of community was also identified. Most user comments were associated with positive attitudes towards exercise during pregnancy and the influencer. However, attitudes towards the types of exercise the influencer performed were mixed (aerobic and body weight exercises were positive; resistance-based exercise with weights were negative). Finally, the online community perceived by users was mostly positive and recognised for offering social support and guidance.

**Conclusions:**

User comments imply resistance-based exercise with weights as unsafe and unnecessary when pregnant, a perception that does not align with current best practice guidelines. Collectively, the findings from this study highlight the need for continued education regarding exercise during pregnancy and the potential for social media influencers to disseminate evidence-based material to pregnant women who are highly receptive to, and in need of reliable health information.

**Supplementary Information:**

The online version contains supplementary material available at 10.1186/s12889-023-15457-6.

## Background

Exercise during pregnancy is associated with pre-, peri-, and post-partum health benefits for both mother and child [1, 2]. Despite comprehensive guidelines for exercise prescription during pregnancy [3], few pregnant women achieve adequate exercise for optimal health benefits [4–7]. Women report a range of factors that influence their engagement in physical activity during pregnancy, including physical discomforts of pregnancy, concerns about the safety of exercise, lack of motivation and/or confidence, lack of social support, and limited access to pregnancy specific exercise resources and programs [8].

Pregnancy is a time that prompts women to consider their health and associated health-related behaviours [9]. As such, this ‘health event’ may result in women adopting healthier behaviours in an attempt to improve their pregnancy outcomes [9]. To help guide these behaviour changes, pregnant women are increasingly sourcing information about exercise, nutrition, and gestational weight gain [10–12] from highly accessible online sources, including social media platforms such as Facebook and Instagram [13].

Social media has the potential to influence individual health [14, 15]. Whilst some users may actively seek out sources of information via social media platforms, others may also be exposed to information they have not explicitly sought out as a result of highly complex platform algorithms that analyse a myriad of user characteristics and online behaviours in an attempt to ‘feed’ users with highly relevant content. Although social media platforms are largely unregulated and can contribute to unhealthy behaviours due to the unintentional and intentional spread of misinformation [15], women’s use of social media has been found to be associated with increased confidence and engagement in exercise, independent of the quality of the information provided [13]. Pregnant women also use social media platforms for advice, social support, and connection with other women going through the same experience [12, 16, 17]. As a result, social media platforms have become important information sources for pregnant women, and have the potential to positively influence attitudes and practice of exercise and other health behaviours [12, 17, 18].

Increased demand and use of social media for information, support, and connection has also given rise to the phenomenon of ‘social media influencers’. A social media influencer can be described as a new genre of celebrity, an ordinary person who has gained fame and influence by sharing content (e.g., videos, photos, inspirational words) across social media platforms [19]. These platforms enable social media users to view, share, and interact with content posted by social media influencers’ via posting of comments (written and visual text) or reacting to posts with emotive icons (emojis) associated with recognised and established emotions, such as ‘like’, ‘love’, ‘care’, ‘sad’ and ‘angry’ [19]. It is common for social media influencers to be paid by companies to promote and/or endorse products [20], and social media ‘fitness influencers’ promote values or lifestyles such as health, physical activity, or wellbeing [21]. This study focussed on Facebook fitness influencers—henceforth referred to as ‘influencers’.

Several recent studies highlight the ability of some influencers to promote and impact health behaviours and intentions such as healthy eating and exercise [20, 22–25]. Influencers can provide visually appealing content and may be perceived as ‘experts’ by their audiences [26]. Influencer credibility is often determined by factors such as success, attractiveness, trustworthiness, and relatability rather than use of academic literature or evidence of relevant influencer qualifications [20, 24]. In fact, research suggests that the more ‘physically attractive’ an influencer is, the greater the perceived credibility and expertise of the influencer, which in turn increases audience engagement and respect for the influencer’s content [27]. Moreover, users can develop parasocial relationships with influencers whereby users experience a sense of connectedness and express feelings of affection, encouragement, gratitude, and loyalty toward the influencer [25]. These parasocial relationships then have the potential power to also influence user behaviours, as users perceive influencers as role models.

Despite influencer content being often underpinned by marketing tactics, such as the use of persuasive communication and product endorsements [24], research suggests that users view information provided by influencers as just as trustworthy and credible as information they receive from family and friends [28]. This is likely because influencers are perceived as ‘everyday people’ who are more ‘relatable’ to users, therefore users consider influencers more trustworthy and credible [28]. Thus, if the influencer is perceived as credible, users are more likely to accept the advice provided and attempt to mimic the influencer’s behaviour [22]. As such, influencers may play a role in promoting health behaviours [14], and are therefore potentially well positioned to target specific population groups such as pregnant women [29, 30]. In fact, approximately 30% of women of childbearing age (between 18 and 35 years) regularly access influencer content [31].

There are several influencers who promote exercise during pregnancy. However, little is known about attitudes toward influencers and their messaging of exercise-related information. Since attitudes are an important determinant of exercise behaviour [32], and influencers have the potential to improve pregnant women’s engagement in exercise, this mixed-methods study aimed to: [1] explore user attitudes toward the influencer and the information provided, and [2] examine user engagement with information related to exercise during pregnancy (posted by influencers).

## Methods

This study adopted a mixed-methods approach. Archival, user-generated data was collected from influencer posts on the social media platform Facebook between August and September 2020, and descriptively and thematically analysed. Facebook was chosen over other social media platforms, as at the time of the study, Facebook held the overwhelming market share of all social media platforms (Facebook 71.7% compared to Twitter at 8.99% and Instagram 7.48%) [33]. Additionally, video posts were chosen as the primary data source since Facebook video content is known to be more engaging than other digital content formats [34]. Ethical approval was obtained from the Central Queensland University Human Research Ethics Committee prior to commencement of the study (Approval number 2020-063).

## Data selection

No previously published protocol was identified to inform data selection protocols for the present study. Hence, researchers experienced in internet and social media research were consulted and a novel two-stage data selection process was developed based on: [1] selection of influencers, and [2] selection of Facebook video content.

### Selection of influencers

For the purpose of the present study, we defined an influencer as one who met the following criteria: [1] ‘Public Figure’ Facebook page, [2] minimum of 10,000 Facebook followers thus including both micro (10,000–50,000 followers) and macro (+ 50,000 followers) influencers [35], [3] was pregnant within the last five years, and [4] minimum of two videos posted on Facebook showing the influencer exercising while pregnant. Two independent researchers (MK & MH) manually searched Facebook and the internet (using Google) to identify a list of female influencers (N = 26).

One researcher (MK) then screened the list of influencers for eligibility, based on these predetermined criteria. Of the 26 potentially eligible influencers, five met the inclusion criteria for the present study. See Table 1 for additional Influencer characteristics.

### Selection of Facebook video content

Video posts were eligible for inclusion if the following criteria were met: [1] video content was posted within the last five years; [2] video content showed the influencer exercising while pregnant; and [3] the video post had a minimum of five user comments (including written and visual text). A minimum user comment limit was set to ensure a level of engagement with the video post and that a variety of comments would be available for analysis. Eligible video posts were then ranked in descending order of user engagement (calculated by totaling the number of emotive icon reactions, shares, and user comments on each post). The two videos with the highest engagement from each influencer were selected for data extraction.

To identity potential video posts for inclusion in this study and limit the potential impact of personalization algorithms affecting the selection of video posts, two independent researchers (MK & MH) conducted a systematic search of the selected influencer’s Facebook pages using combinations of the following search terms: *pregnant*, *pregnancy*, *exercise*, and *fitness*. All results were screened for eligibility.

## Data extraction

**Fig. 1:**
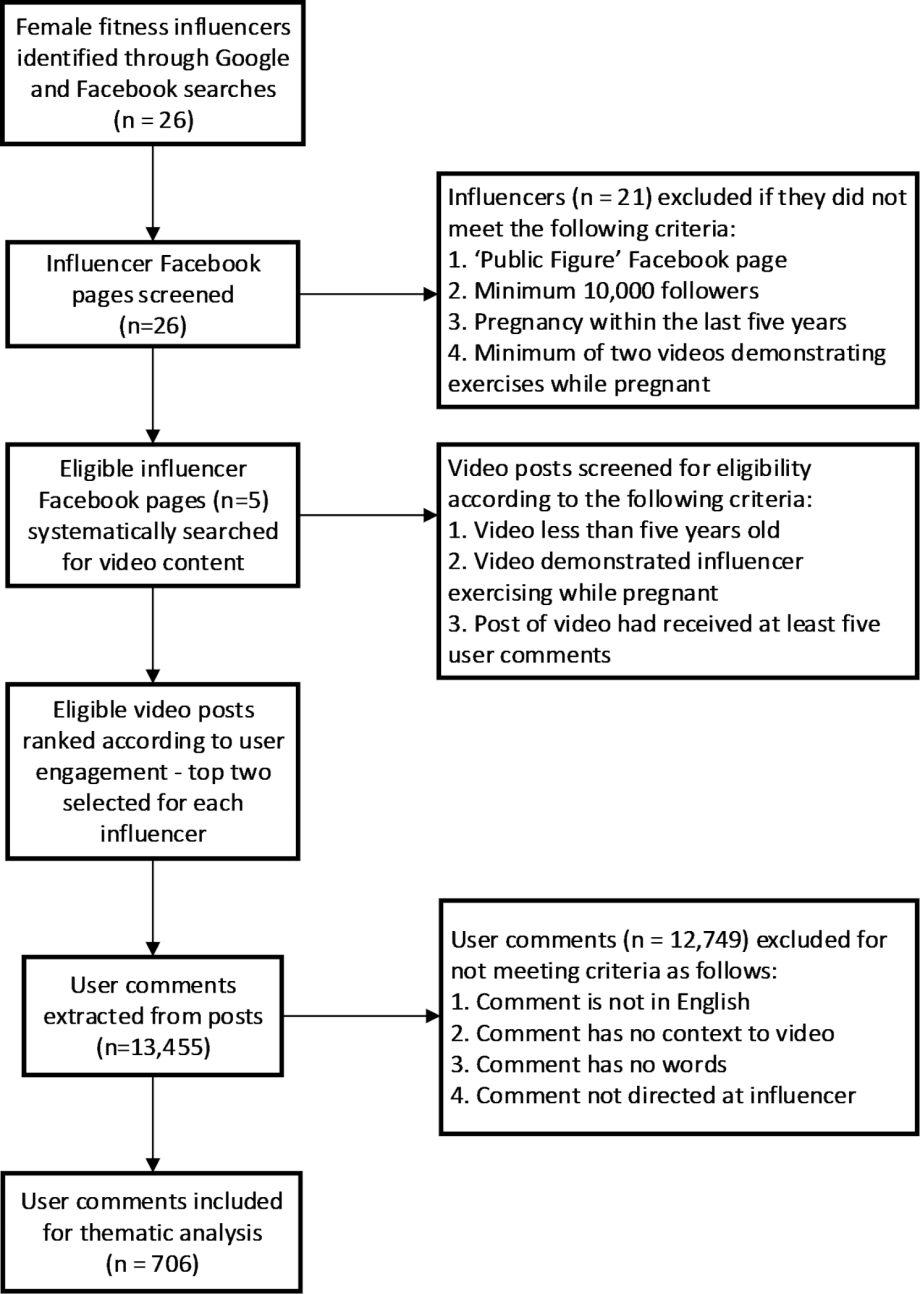
Flowchart outlining the process of data collection

The final selection of video posts (*n* = 10) was assigned to nine independent researchers (MH, SC, KLA, CH, SK, SS, LH, CG, SA) for data extraction and screening. Eight researchers were allocated one video post each, and one researcher was allocated two posts (due to the low number of user comments present on each post). Each researcher was provided with a hyperlink to their assigned video post, a pre-formatted Microsoft Excel template to record the data, an instructional data extraction video, and written instructions outlining the process of accessing, extracting, recording, and screening the data [see Additional file 1]. Figure 1 outlines the process of data collection.

The template recorded the following information from each included Facebook video post: hyperlink, influencer’s name, date of data extraction, type of exercise/s used in video, gestational age (where available), and any written commentary uploaded with the video post. Engagement data was also extracted, including the number of times the video was shared, the number of views the video had received, and the type and number of emotive icon reactions generated.

Public user comments attached to each post were extracted by manually copying and pasting comments from the video post into the excel template. Extracted comments were screened using the following exclusion criteria: [1] non-English language comments, [2] comments with no context (such as tagging another Facebook user), [3] visual comments with no words (e.g., comment only included a photo, symbol, emotive icon reaction, and/or graphic) as the true meaning of visual comments without further context cannot be determined with any level of accuracy, and [4] comments that were not directed at the influencer, or related to the video content, exercise, or pregnancy. Importantly, interactions between the influencer and users were not explored, as this was outside of the scope of this study. Remaining data were then collated in a separate Excel spreadsheet and de-identified (influencer and commenter names were removed) in preparation for analysis.

### Data analysis

An analysis of user interaction with posts on social media was conducted to provide insight into level of engagement with the content [36, 37]. For quantitative analysis, summed totals for each of the seven emotive icon reactions (like, love, care, haha, wow, sad, angry) were calculated across the selected Facebook video posts. Qualitative analysis of user comments was achieved using an inductive thematic approach, in accordance with Braun & Clarke’s [38] six-step process: [1] data familiarisation, [2] initial code generation, [3] theme identification, [4] theme review, [5] theme definition and naming, and [6] report production.

Two reviewers (MK & TT) independently screened and coded the first 100 comments to generate initial codes, before coming together to review and discuss. The coding process was then completed in full by the first reviewer, with codes continually modified and refined during the process. The second reviewer was consulted during and at completion of coding until a consensus was reached, and a final codebook developed [see Additional file 2]. Codes were categorised to identify preliminary themes and sub-themes by the first reviewer, and the second reviewer was consulted to discuss and review the themes. Codes and preliminary themes were validated independently by two additional reviewers (MH & SW). Preliminary themes were then categorised into major themes until final overarching themes and sub-themes were identified.

## Results

Four of the five influencers’ posts included in this study were linked to some sort of advertising, marketing or sponsorship. Two influencers included a hyperlink to their fitness app in both posts, while one influencer included references to the clothing the influencer was wearing. Another influencer included their Facebook page address in their two posts while the remaining influencer made no reference to any advertising, marketing or sponsorship in either of their posts. None of the influencers declared that their posts were sponsored, or that they had received any form of renumeration from an external source for any of their posts included in this study. See Table 1 for additional influencer characteristics below.

At the time of data collection, the 10 selected video posts had been viewed a total of 12,117,200 times and were shared a total of 11,181 times. The total number of comments (including written and visual text) from each post ranged from at least 8 comments to 8,300. After removing comments that only consisted of visual text (emojis), written text comments ranged from 1 to 251. The number of emotive icon reactions generated by all video posts totalled 128,804, with the most frequently used icon being ‘*like’* (81.48%). Detailed Facebook user engagement information is shown in Table 2.

A range of exercises including aerobic exercises (such as skipping and modified burpees), resistance-based exercises (consisting of either bodyweight exercises [squats, lunges, planks]) or exercises that encompassed weights (shoulder press, deadlift, squats with a kettlebell), were demonstrated in the video posts by the influencers. Some video posts included a combination of aerobic and resistance-based exercises.

A total of 706 Facebook user comments were thematically analysed, and four overarching themes identified: [1] attitudes towards exercise during pregnancy, [2] attitudes towards the influencers, [3] attitudes towards the types of exercise, and [4] community. The first three themes characterise both positive and negative views, while the final theme, community, relates to the level of social support that was apparent among Facebook user comments. See Table 3 for a summary of themes and subthemes.

Attitudes towards exercise during pregnancy in general, and attitudes towards the influencer were mostly positive. However, attitudes towards the types of exercise the influencer performed were mixed, with all negative comments found to be associated with posts that involved the influencer performing resistance-based exercises using weights.

**Table 1:** Influencer Characteristics ^a^ Total influencer followers according to Facebook as of 20 January 2023

Influencer #	Followers ^a^	Page Category	Facebook Intro	Stage of pregnancy	Exercises within posts	Advertising / marketing / sponsorship
1	28,000 000	Fitness Trainer	Join my community of confident, healthy and fit women worldwide!	3rd Trimester	Burpees, squats, bicep curls, side raises, kettlebell high pull	Hyperlink to influencer app
				3rd Trimester	reverse arm curls, resistance band high pulls, medicine ball squats, held/prolonged squat, resistance band donkey kicks’, bicep curls	Hyperlink to influencer app
2	9,800,000	Public Figure	Health & Fitness Expert / Qualified PT	Not disclosed	deadlifts	Hyperlink to influencer app
				Not disclosed	squat, plank push up, sumo squat, fire hydrant (hip abduction), modified push up, clam	Hyperlink to influencer app
3	2,000 000	Public Figure	Bikini Pro, Fitness Model, Certified Personal Trainer, Mom & NYT Bestselling	Not disclosed	skipping, medicine ball, weights, battle rope, static row, boxing	Influencer FB address
				Not disclosed	weighted squats, kettlebell swing, jump rope	Influencer FB address
4	125,000	Athlete	Email Instagram Fan mailing address	40 weeks	Shoulder press, slam balls, jumping lunges, triceps extension, speed skaters, reverse fly	NIL
				40 weeks	Sled push, hammer chops on tire, ball toss over the shoulders, lawnmower pulls, push-ups, Russian kettlebell swings	NIL
5	38,800	Athlete	“[Influencer Name] Official Facebook Page” Instagram	13 weeks	parallel rows, pistol squat to reverse lunge, plank walks	Clothing brand
				14 weeks	resistance band hack squat, squat to curtsy	Clothing brand

**Table 2:** Number of views, shares, comments, and emotive icon reactions per Facebook influencer video post

Post #	Views	Shares	User comments – before screening ^a^	User comments - after screening ^b^							Emotive icon reactions								
					Like (%)		Love (%)		Care (%)		Haha (%)		Wow (%)		Sad (%)		Angry (%)		Total emotive icon reactions per post
1	5,500,000	6,208	8,300 ^a^	251	47,000	79.1	8,200	13.8	2	0.2	119	0.2	4,000	6.7	33	0.1	53	0.1	59,407
2	6,300,000	4,400	4,300 ^a^	180	48,000	85.0	6,500	11.5			76	0.1	1,800	3.2	30	0.1	54	0.1	56,460
3	18,800	88	302	104	2,000	72.2	390	14.1			1	0.0	368	13.3	2	0.4	11	0.4	2,772
4	239,000	233	214	72	4,700	81.3	862	14.9	138	2.4	4	0.1	76	1.3	1	0.0			5,781
5	28,700	158	175	39	1,800	74.5	527	21.8			1	0.0	80	3.3	4	0.2	4	0.2	2,416
6	16,700	51	63	31	1,100	78.9	246	17.6			4	0.3	44	3.2					1,394
7	1,900	12	47	16	67	69.8	21	21.9				0.0	8	8.3					96
8	3,300	20	36	10	65	61.9	30	28.6			1	1.0	9	8.6					105
9	4,400	5	9	2	108	45.8	108	45.8			4	1.7	16	6.8					236
10	4,400	6	9	1	111	81.0	21	15.3					5	3.6					137
Totals	12,117,200	11,181	13,455	706	104,951		16,905		140		210		6,406		70		122		

^a^ Facebook restricts the free export of user comments to a maximum of 500, hence all comments for this post were unavailable to be included for screening.

^b^ All user comments were screened to remove visual texts and identify written texts for further thematic analysis.

**Table 3:** Themes and subthemes resulting from thematic analysis of Facebook user comments

Theme	Subthemes
Attitudes towards exercise during pregnancy	Health benefits to mother and baby
	Exercise is beneficial and safe when pregnant
	Exercise is unachievable
Attitudes towards the influencers	Motivational and inspiring
	Relating to the influencer
	Comparison to self
	Expertise and credibility
Attitudes towards the types of exercise	Not all exercise is safe
	Expertise and credibility
Community	Social support and guidance

### User attitudes towards exercise during pregnancy

#### Health benefits to mother and baby

Users generally held positive attitudes about the importance of exercising when pregnant and acknowledged the health benefits for the mother and child. Users identified improved mental health and mood, reduction in the physical discomforts of pregnancy, reduced labour complication, and healthy development of the baby as benefits of exercise during pregnancy. For many users, exercise was recognised as contributing to a quick and safe labour:

*You’ll find that labour is so much “easier” when you’ve been fit before and during your whole pregnancy!! It gives you the endurance you need! With my first I ran 3 miles on my due date!*

Users also acknowledged that exercise behaviours of the pregnant woman supported foetal development:

*Working out at moderate intensity is actually proven to be more beneficial. Increased blood, oxygen, and endorphins to the foetus help strengthen baby and actually help build a healthier heart in the baby.*

#### Exercise is beneficial and safe when pregnant

Many users considered exercise during pregnancy to be an important factor in prenatal care, with one user suggesting:

*…it is encouraged by doctors to exercise and maintain a healthy lifestyle during pregnancy.*

Some users even sought advice directly from the influencer or other commenters, recognising the importance of exercise during pregnancy, and highlighting the perceived ‘expert role’ of influencers:

*…can I ask you what kind of exercises you did during your pregnancy? I know exercising during pregnancy is good, but I know there are specific types of exercises to avoid as they are harmful to the baby. I just wanted to know which exercises were safe.*

Many users alluded to the need for exercise during pregnancy to be ‘safe’ and be guided by healthcare providers, and that users should modify exercise and/or “listen” to their body. When the influencer was performing resistance-based exercises with weights and aerobic exercises, one user commented:

*[Influencer] is well conditioned to this exercise before her pregnancy and is using safe regressions to her training, at a very safe time in her pregnancy to be doing this training.*

#### Exercise is unachievable

While users had positive attitudes about the influencer and the exercises depicted, they often perceived the exercises performed by influencers as unachievable for themselves and made comparisons between the capabilities of the influencer and themselves. A commonly expressed attitude by users towards the influencers was ‘she can, but I can’t’. Users also expressed negative beliefs and doubts about their own ability to partake in exercise when pregnant, due to past or current pregnancy experiences.

Comparative comments were often linked to factors that prevented users from exercising such as, concerns about risk, lack of motivation, negative social influence, physical discomforts of pregnancy, health complications, lack of time, lack of knowledge, and fatigue.

*My second pregnancy in a row I would have been totally burned out after that far along! I was exhausted.*

*I didn’t work out before or during my pregnancy. Even though I started under 130 lbs and only gained just under 30 lbs, I had some issues with feet and ankle swelling and my son was born early. I wish I was in better shape beforehand!*

*…I puked 24 hrs a day 7 days a week for 8 months. I barely had the energy to walk to the bathroom so there wasn’t any exercise for me...except for vomiting...lol*

Overall, users held positive attitudes about the importance of exercising when pregnant, acknowledging the health benefits and need for safety when exercising during pregnancy. Users also perceived exercise an important aspect of prenatal care, and some users actively sought guidance from influencers. Some users also perceived the exercises of the influencer to be unachievable for themselves, resulting in comparisons between the capabilities of the influencer and themselves. These perceptions were often accompanied with personal user experiences often linked to factors that prevented the user from exercising.

### Attitudes towards the influencer

#### Motivational and inspiring

Facebook users’ attitudes toward the influencer were typically positive, with influencers viewed as role models. The inspirational role of the influencer was characterised by comments providing praise and admiration:

*I’m almost 30 weeks and absolutely love following your workouts! You been an inspiration and this being my first pregnancy has been wonderful so far! A lot of it has to do with me staying so active! Thanks for your posts!*

Synonymous with expressions of praise and admiration were comments about the influencer’s physical attractiveness, strength, capabilities, and commitment to exercising while pregnant:

*Your dedication, positivity and beauty motivate and amaze me!*

Other users aspired to achieve the influencers level of fitness and the influencers physique:

*I wish I looked like her, she is so fit!*

#### Relating to the influencer

Intertwined with user admiration for the influencer was a positive (albeit one-sided) relationship with the influencer. This one-sided parasocial relationship was portrayed when users expressed knowledge of intimate details about the influencer’s life (e.g., living location, children or partners’ names), aggressively defended the influencer against criticism from other users, gave personal well wishes to the influencer and their family, or found other ways to relate directly with the influencer:

*Congratulations, I know how you feel am just over the 26 week mark, still training but nowhere near what I use to do and I can’t wait to get back at it probably as it’s doing my head in not been able to lift my normal strength in weights lol .. x*

#### Expertise and credibility

Influencers were mostly trusted and regarded as experts by users. This perceived expertise also meant users saw the influencers as credible sources of information. Some users commented, “She knows what she’s doing’, and “Her form is perfect and it is not like she is a beginner exerciser”, while others sought advice:

*Did you have a pregnancy workout daily routine we can follow? I am currently 22 weeks. I’ve never seen a modified burpee before! This is great! I have some joint issues and could totally do this version! Thanks for sharing.*

Overall, users’ attitudes toward the influencer were positive. Users considered the influencers trustworthy and credible. Comments mostly consisted of praise and admiration in addition to positive comments regarding the influencer’s physical attractiveness, strength, capabilities, and commitment to their exercise behaviours. Evidence of parasocial relationships between the user and the influencer emerged as users demonstrated connections with the influencer (e.g., citing personal details about the influencer), and defended the influencer behaviours to other users’ criticism.

### Attitudes toward the type of exercise

#### Not all exercise is safe

Although users expressed a desire to exercise, uncertainty about the safety of exercises during pregnancy was another barrier to performing the exercises themselves:

*I’m pregnant now and would love an easy list of what’s safe and when, so I can feel more confident in the exercise I do.*

Users held mixed attitudes towards the different types of exercise the influencer performed. When influencers engaged in aerobic or bodyweight exercises, user attitudes towards the influencer and the type of exercise the influencer was performing were positive and supportive. An example comment relating to these types of exercises was:

*YOUR body is MADE for burpees, so don’t sweat it. You kept doing what your body was used to.*

In contrast, when influencers engaged in resistance-based exercise using weights, the user attitudes were mixed. Users expressed a range of concerns and questioned the safety and necessity of the exercise being performed:

...*is that a good idea to be lifting heavy weights when you are this far along?*

Other users perceived exercises involving weights to be high risk, harmful to the baby, and contradictory to health provider recommendations:

*Any midwife or gyno doc will tell you don’t lift or strain during last trimester. Why risk both your lives.*

Specifically, almost all negative comments from users towards influencers were associated with the influencer engaging in resistance-based exercises using weights. Medical complications during pregnancy, and the advice of healthcare providers, were identified as some of the reasons why resistance-based exercises using weights was viewed as unsafe:

*I was absolutely huge out front and could NOT do any of these sorts of exercises whilst pregnant. Believe me I so wanted to, but genetics had other plans for me. I couldn’t even see a weight on the floor past the bump let alone pick it up. It would have been incredibly detrimental to me to have put any more strain on my abdominals. My midwives checked my abdomen throughout my pregnancy and strongly advised against anything like this. I ended up with a 6cm separation post-partum, so they were completely on the money.*

#### Expertise and credibility

Negative user attitudes towards resistance-based exercises using weights during pregnancy was also associated with discreditation of the influencer’s status as an exercise professional and/or expert. Although some users appeared to admire an influencer, the same users also disapproved of the influencer’s choice in exercise:

*You are an amazing and smart person and a true inspiration to all out there, but this is too risky sorry.*

Influencer credibility was also brought into question after they chose to perform resistance-based exercise using weights when pregnant, as it was perceived to be against healthcare provider recommendations and exercise guidelines, as one user commented:

*I am under the impression that when you reach a certain point in pregnancy, you shouldn’t be lifting weights. My OBGYN told me I couldn’t lift weights but could do other exercises.*

The influencer’s motivations to exercise was also questioned and deemed as a selfish act of choosing appearance over health:

*…exactly figure or health of baby. Totally irresponsible.*

Overall, user attitudes were positive and supportive when influencers engaged in aerobic or bodyweight exercises. but users expressed concerns about the safety and need when influencers engaged in resistance-based exercises. Negative user attitudes tended to lower levels of influencer trust and credibility, with some users questioning the influencers expertise.

### Community

#### Social support and guidance

A ‘community’ theme was identified however this theme is not directly related to attitudes about exercise during pregnancy. This theme suggests that by sharing personal experiences, seeking and offering advice, and supporting other users, a supportive community of users is created independent of the influencer. Within this community, users challenged attitudes and misconceptions about exercise during pregnancy and offered emotional support and guidance about exercise to each other:

*[user name] that’s how I was with my first. I knew that with my second pregnancy, I wanted to change that. If you have another baby in the future, definitely try to work out before and during! Makes a world of difference.*

*[user name] congratulations you’re a wonderful woman, just like me, I have 7 beautiful children, 4 boys and 3 girls...hope that you’re in good health, because it’s all that matters.*

*[user name] we live in a country with extremely poor habits. Exercise is one of the keys to long healthy life and very important while pregnant.*

Overall, a supportive community of users was created independent of the influencer. Users shared personal experiences, sought advice from each other and offered emotional support and guidance to one another. Attitudes and misconceptions about exercise during pregnancy were also challenged between users.

## Discussion

The primary aim of this study was to explore attitudes towards influencer-posted pregnancy exercise content on social media. Various user attitudes were identified, including those towards exercise during pregnancy, the influencer, and different types of exercise. Positive and negative attitudes towards exercise during pregnancy emerged from the analysis, with acknowledgement of the positive health benefits contrasting with negative beliefs that exercises demonstrated by influencers would be unachievable for the average pregnant woman. Attitudes towards influencers were generally favourable, with users viewing them as motivational, relatable, and credible. However, attitudes towards the types of exercise performed by pregnant influencers displayed a degree of dissonance, with users perceiving aerobic or bodyweight exercises as safe for mother and child, but resistance-based exercise using weights as concerning, unnecessary, or unsafe. An additional theme of ‘community’ was also identified, highlighting the social networking/support aspect of social media platforms as found in previous studies [12, 17].

Facebook users generally expressed positive attitudes towards exercise during pregnancy. Users recognised the health benefits of exercise for the mother and baby, the importance of maintaining a level of activity during their pregnancy and expressed a desire to increase their exercise behaviours. These results are similar to findings of previous studies which report that women believe some form of exercise is essential to engage in when pregnant and beneficial for labour, mother, and child [8]. While evidence of positive attitudes towards exercise is a starting point for influencing pregnant women’s intentions to exercise [32], previous research findings of low participation in exercise during pregnancy suggests that positive attitudes alone are not sufficient to change the exercise behaviours of pregnant women [8].

Despite recognition of the importance and benefits of exercise during pregnancy, users identified several barriers to engaging in exercise similar to previous studies [8, 39]. Physical discomfort, health/pregnancy complications, lack of motivation, time constraints, limited availability of credible information, negative attitudes of family and friends, and concerns about the risks of harm to themselves or their unborn child were all identified as barriers to exercise during pregnancy. In this study, users also perceived the exercises performed by influencers as unachievable for themselves and made comparisons between the capabilities of the influencer and themselves [8, 39]. This contrasts somewhat with previous research that suggests the ability of an influencer to perform difficult or advanced exercises while pregnant inspires some users to engage in similar exercises [40]. Our findings demonstrated positive attitudes of trust in the influencer’s knowledge, with users generally perceiving influencers as having expertise in exercise during pregnancy, and commenting about the inspiration and motivation they drew from the influencer. According to the literature, perceived expertise and trustworthiness of the influencer [28] as well as personal relevance of the message and the user’s affective attitudes [41] can have a positive effect on users’ attitudes towards the influencer [20] and can also result in users being persuaded to make behaviour changes, at least in the short term [22, 41]. Further to this, we identified the presence of parasocial relationships where influencers were perceived by users as someone they felt personally and emotionally connected to and trusted as credible sources of advice about exercise during pregnancy [25]. As such, the influencer may be seen as a role model whereby users adopt exercise behaviours promoted by the influencer through the perceived trust and credibility the user feels for the influencer [25]. This was evidenced in our study by user comments that reflected praise, admiration, vehement defence of the influencer, and the suggestion of a one-sided relationship with the influencer. This combination of a positive attitude and emotional attachment to the influencer, expertise and trustworthiness, influences a person’s desire to mimic the behaviours of the influencer [22]. Therefore, pregnant women may be motivated to increase their engagement in exercise by watching positively perceived influencers exercising when they are pregnant [21].

Although influencers were perceived as experts by many, when they performed resistance-based exercises using weights (as opposed to aerobic or bodyweight exercises), their perceived expertise and credibility came into question. This attitude is consistent with literature which suggests that when influencers are not seen as trustworthy or knowledgeable, they lose credibility with their audience [24]. According to current guidelines, it is recommended that pregnant women engage in two sessions a week of muscle-strengthening resistance-based exercises at moderate intensity using bodyweight, resistance bands or light weights [42]. Despite evidence to the contrary, some users in the current study viewed resistance-based exercises using weights as a dangerous behaviour, risking harm to the mother and unborn child. Thus, with an influencer advocating for exercises perceived as unsafe to be performed during pregnancy, users can perceive them as not credible. Findings from this study are similar to previous reports which suggest that while many women understand and acknowledge the safety and health benefits of some types of exercise during pregnancy, there remains a segment of the population who are uncertain [2, 43]. Based on these current and past findings, it appears that women either do not understand the different types of resistance exercises, are not fully aware of the recommended guidelines for exercise during pregnancy, or may be receiving outdated and misguided information from healthcare professionals [44, 45].

In addition to seeking information from influencers about exercise during pregnancy, it was apparent that users in this study also experienced a sense of community and networking, using the platform as an opportunity to seek support and advice from other users, and to share common experiences. Social media is an accessible form of social support [18] that enables women to learn from each other [46]. Access to social support in like-minded communities may also reinforce social norms about exercise, and encourage women to participate in exercise during pregnancy [39, 47].

Findings of the current study indicate that the misconceptions about pregnancy safe exercises may be a contributing factor to some of the barriers that women experience, and the negative attitudes they hold about their ability to engage in exercise while pregnant. Access to credible information affects both the attitudes towards exercise during pregnancy and a pregnant woman’s perceived behavioural control, which is the confidence, resources, and ability they have to overcome the barriers they experience [48, 49]. Information about the risks, benefits, and prescription of exercise during pregnancy could improve the confidence of pregnant women, contribute to their sense of control to engage in exercise [49], improve attitudes towards exercise [32], and overall, encourage women to participate in exercise [40]. Advice received from healthcare providers about exercise during pregnancy is often conservative, likely owing to a lack of understanding or awareness on the part of the health professional [45, 50], and may not include information about the safety and efficacy of resistance-based exercise using weights [51]. This highlights an urgent need to equip healthcare providers with the knowledge, resources, and confidence to provide accurate exercise advice to encourage pregnant women to exercise [45, 50, 52]. Findings of the current study also add to the existing research that more health interventions are needed to address the information gaps about exercising during pregnancy [51], and that influencers and social media platforms may provide a possible avenue for health promoters to access wider audiences.

## Limitations

To the best of the researchers’ knowledge, no other study has examined attitudes towards Facebook influencers targeting exercise during pregnancy. Previous studies have generally focused on attitudes towards exercise during pregnancy with women in offline environments [8]. Some studies have focused on exercise attitudes online [21]; however, none are specific to exercise during pregnancy and influencers. This study has some limitations which need to be highlighted.

During data extraction and screening, users were deidentified, and demographic information was not collected, hence limiting the extent to which the findings are representative of pregnant women’s attitudes. Furthermore, due to the anonymity of the data, cultural backgrounds were unknown. Therefore, the generalisability of the findings and diversity across cultures is unknown. Another limitation is the choice of only one social media platform for data collection. Despite some Facebook posts having several thousand comments attached, access to the data was limited (Facebook only permits free export of 500 user comments), and only a small portion of these could be extracted for analysis. Other factors to consider when interpreting the findings include the potential presence of ‘online trolls’ who purposely leave negative comments on social media sites and the possibility that influencers may have purchased comments to boost their status and performance metrics and reach, as these factors could not be controlled for in the present study. Finally, although our findings are consistent with other studies exploring attitudes toward exercise during pregnancy, they may be confounded by sampling bias. Some comments may have come from users who were actively seeking out exercise related content online through influencers, or by users who were selectively exposed to the posts due to platform algorithms based on their prior online behaviour. Nonetheless, these users are likely to share common characteristics or behaviours, hence the reason they landed at the same content, and therefore may share similar attitudes toward the influencer. Conversely, it is possible that users with no interest in exercise related content or conflicting attitudes were not exposed to the post, and therefore were unable to provide any commentary for consideration.

## Directions for future research

Very little research exists exploring the potential impact and/or influence of social media influences on exercise behaviours among pregnant women. Future studies could investigate the demographics of women who follow influencers, and how and why pregnant women engage with influencers and also other users, as this information might provide insight into how information is shared with other social networks and could also be used to develop strategies to better engage women with evidence-based information. Similar studies could also be extended to other social media platforms such as Instagram and YouTube or other specific platforms that women use to follow influencers. Moreover, future analysis could extend beyond user comments to also include the interaction between users and influencer, as influencer interaction may affect user engagement and user trust.

## Conclusion

By examining the attitudes towards influencers and exercise during pregnancy on Facebook, this study found that users recognise the health benefits and importance of exercise during pregnancy. Additionally, the present study shows that women are seeking information and advice from social media influencers, who for the most part, are trusted and perceived as experts. However, despite exercise guidelines recommending that resistance-based exercise using weights is safe and beneficial during pregnancy, the perception still remains that these exercises are unsafe and unnecessary. The popularity of social media may provide a unique platform for evidence-based information dissemination. Further, health promoters should explore opportunities to engage with influencers, thus utilising their reach, perceived credibility, and expertise to disseminate evidence-based information to highly receptive and engaged audiences and positively influence the exercise behaviours of pregnant women.

## Electronic supplementary material

Below is the link to the electronic supplementary material.

Supplementary Material 1

Supplementary Material 2

## Data Availability

The datasets analysed during the current study are available from the corresponding author on reasonable request.
