# Supplementary material for: A mixed-methods exploration of attitudes towards pregnant Facebook fitness influencers
Source: BMC Public Health. 2023 Mar 27;23:569. doi: 10.1186/s12889-023-15457-6 (PMC10041693; doi:10.1186/s12889-023-15457-6)
Supplement: Supplementary file 1 — Supplementary Material 1 [file 12889_2023_15457_MOESM1_ESM.docx]

**Additional file 2: Thematic code book**

| **Code** | **Definition** |
| --- | --- |
| Admiration and praise | Admiration, praise. inspiration towards the influencer. High regard for the influencer. |
| Support and encouragement | Approval of influencer actions by offering support and encouragement |
| Appearance | Attractiveness, physical appearance, and aesthetics of the influencer |
| Shared experience | Users shared similar experience of pregnancy or exercise with the Influencer, relating to the influencer |
| Defends the influencer | Defends actions of the influencer to other commenters. |
| Personal connection | Emotional connection to the influencer and emotional investment in life of influencer (e.g., a personal joke, well wishes, comments directed at their personal life and family), |
| She knows what she is doing | Influencer is an expert with a high level of knowledge to exercise safely; she knows her body best. |
| Influential content | Aspirations or intent to copy the influencer; praise or questions about the content (work outs programs offered). |
| Health benefits | Acknowledges benefits of exercise for pregnancy/childbirth/breastfeeding. |
| Community | Not directed at the influencer. Seeking advice and offering advice and support to other users and sharing experiences with each other. |
| She can but I can't | Comparing abilities with the influencer |
| Barrier | Personal barriers that prevent exercise |
| Influencer lacks credibility | Disapproval, judgement and questions about the influencer’s actions, knowledge and experience about exercise and pregnancy. |
| Exercise is unsafe | Concerns for the safety of the exercise and risk of harm to mother and unborn baby. |
| Disapproval | Negative comments, a dislike or insult towards the influencers |
| Program queries | Questions or comments specific to the influencer’s programs. |
| Disbelief | Disbelief that influencer is pregnant and/or exercising while pregnant |
| Pregnancy advice | Advice directed at how the influencer should behave during their pregnancy out of care or concern |
