# Supplementary material for: A mixed-methods exploration of attitudes towards pregnant Facebook fitness influencers
Source: BMC Public Health. 2023 Mar 27;23:569. doi: 10.1186/s12889-023-15457-6 (PMC10041693; doi:10.1186/s12889-023-15457-6)
Supplement: Supplementary file 2 — Supplementary Material 2 [file 12889_2023_15457_MOESM2_ESM.docx]

**Additional file 1: Instructions for Facebook data extraction**

The spreadsheet contains three worksheets named used to record the data and examples of this.

- 1_Post details,
- 1_Raw Data
- 1_Comments Screened (each person will have a different number).

Instructions for each worksheet are provided below:

Post Details

This is partially completed, and the remaining details to be extracted are as follows:

1. Open the link provided and record the following:
2. Date of data extraction
3. Exercises: record the exercise shown in the video. This may also be written in the post description. If unknown, please make a note and we can help.
4. Total emotive reactions. Press the emoji button at the bottom. This will show the individual totals of each emoji. Press the ‘More’ button to expand the list. Record each total in the spreadsheet.

Extracting the Comments

Comments will be extracted to the spreadsheet **Raw Data**.

1. Open the comments and change from ‘Most Relevant’ to ‘All Comments’
2. Expand all the comments and replies.
3. Ensure you click ‘See more’ on the larger comments.
4. Copy all the comments and record this into the worksheet named Raw Data.
5. Use the option ‘Paste Special’ then select ‘unicode’.

Note: The total number of comments noted on the posts will be less than what you are available to extract. For example, the post may say 800 comments, but you can only access 280 of these.

Screening the comments

1. Raw data/comments are to be screened and exclude any comments based on the below criteria:

- Non-English posts
- Tags with no context
- Comments that only include Emojis / GIFS / symbols / pictures with no comment
- Comments that do not speak / relate directly to the influencer, exercise or pregnancy eg - " you have obviously no idea what you’re talking about" or "hahahaha wrong honey"

1. Highlight (in yellow) the Author and Comments that are identified for inclusion.
2. Copy the Author name and comment over to the Screened Comments worksheet.
3. Delete any emojis within the text of the comment. For example, remove the heart emojis at the end of the comment. As seen in the below comment:
   “U look great! I wish I had that much energy when I was pregnant! ❤️❤️❤️”

Notes: Any comments you are unsure of, please highlight in blue so this can be reviewed.
